# Kidney displaced by giant retroperitoneal liposarcoma in HIV patient

**DOI:** 10.1590/S1677-5538.IBJU.2019.0515

**Published:** 2020-03-25

**Authors:** Sheng-Chen Wen, Chunhsuan Lin

**Affiliations:** 1 Department of Urology Kaohsiung Medical University Hospital Kaohsiung Medical University Kaohsiung Taiwan Department of Urology, Kaohsiung Medical University Hospital, Kaohsiung Medical University, Kaohsiung, Taiwan

## CASE DESCRIPTION

**Figure 1:**
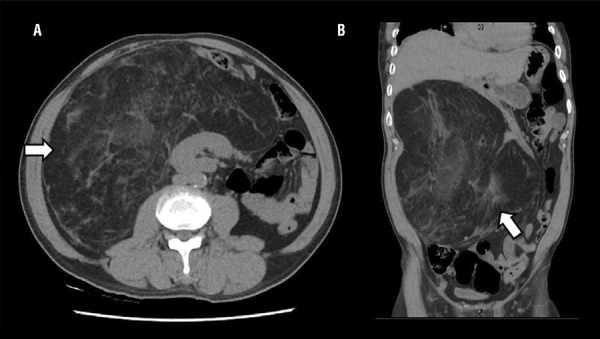
A) Right kidney dislocated in epigastrium by the retroperitoneal component of the mass. B) Right colon displaced against abdominal wall and most of the small bowel in left side of abdomen.

**Figure 2:**
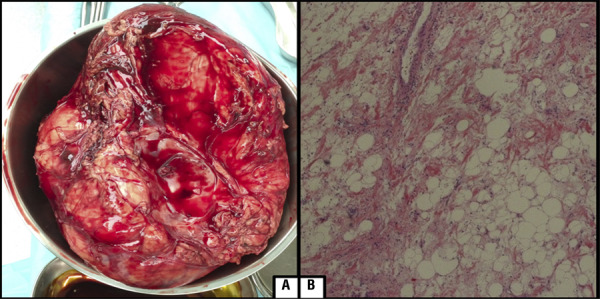
A) Intraoperative image of the resected specimen. B) Histological examination showed presence of atypical, hyperchromatic stromal cells with a varying number of lipoblasts.

A 56-year-old male with a history of infection of human immunodeficiency virus over ten years, was referred to our center because of intermittent epigastralgia and gradual increase of abdominal girth in the last two months. Physical examination revealed palpable abdominal mass at the right upper quadrant measuring around 20cm. Laboratory examinations of complete blood counts, urine tests, and tumor markers were otherwise normal. CT scan of the abdomen showed a huge fatty mass of 23.3 x 22.9 x 34.5cm with mixed density and pathological contrast enhancement arising in the retroperitoneum. The mass displaced right kidney in epigastrium ( Figure -1A ) and most of the bowel away from their natural position in right side of abdomen ( Figure-1B ). Surgical excision of the mass was performed through a para-midline incision, and revealed a giant clearly encapsulated fatty tumor deriving from the right retroperitoneal fatty tissue ( Figure-2A ). The mass was completely extirpated without resection of adjacent tissue or organs. The final histopathological report showed a well-differentiated liposarcoma of the retroperitoneum ( Figure-2B ). The patient’s postoperative course was uneventful and he was discharged on the 6th postoperative day. At one year post-surgery, there was no evidence of recurrence on different CT scans.

Retroperitoneum is the primary site in about 15% of soft tissue sarcomas (STS) ( 1 ). Liposarcomas account for approximately 40% of retroperitoneal sarcomas making them the most common type ( 2 ). The differential diagnoses of masses with retroperitoneal fat content is an usual diagnostic predicament. Computed tomography (CT) imaging features that suggest malignancy include large lesion size, presence of thick septa, presence of nodular and/or globular or non-adipose mass-like areas, and decreased percentage of fat composition ( 3 ). Histopathology is central for the distinguishing workup of lipomatous tumors. In the case that lipomalike well differentiated liposarcoma may be hard to discriminate from lipoma, an immunohistochemical panel composed of MDM2 and CDK4 can be useful ( 4 ).

Infection with the human immunodeficiency virus (HIV) and the subsequent destruction of T4-positive helper cells are associated with the development of various malignancies.

HIV-infected patients may be at greater risk for other forms of cancer because of changes in immune surveillance. In immunodeficient populations, other than Kaposi sarcoma and other sarcoma types, only leiomyosarcoma and angiosarcoma occur disproportionately in these patients ( 5 ). Liposarcomas is usually a malignancy of later life but rare to be encountered in HIV populations. Although it is required to obtain negative resection margins ( 6 ), it is necessary to weigh the benefit of free margin resection against the adversity of medical complication in cases where the tumor invades into a nearby organ.
